# Clinical and Radiological Comparisons of Percutaneous Low-Power Laser Discectomy and Low-Temperature Plasma Radiofrequency Ablation for Cervical Radiculopathy: A Prospective, Multicenter, Cohort Study

**DOI:** 10.3389/fsurg.2021.779480

**Published:** 2022-02-09

**Authors:** Xueqin Lan, Ziyang Wang, Yuzhao Huang, Yuncheng Ni, Yunwu He, Xiaofeng Wang, Chunsheng Wu, Rong Hu, Rui Han, Gangwen Guo, Zhenxing Li, Xuan Zhang, Jianping Zhang, Qin Liao, Dong Huang, Haocheng Zhou

**Affiliations:** ^1^Department of Pain, Institute of Pain Medicine, The Third Xiangya Hospital, Central South University, Changsha, China; ^2^Department of Anesthesiology, The Affiliated Changsha Central Hospital, University of South China, Changsha, China; ^3^Department of Orthopedics, The Third Xiangya Hospital, Central South University, Changsha, China; ^4^Department of Pain, The Second Affiliated Hospital, University of South China, Hengyang, China; ^5^Department of Pain, Hunan Aerospace Hospital, Changsha, China; ^6^Department of Pain, People's Hospital of Xiangxi Prefecture, Jishou University, Jishou, China; ^7^Department of Anesthesiology, The Third Xiangya Hospital, Central South University, Changsha, China; ^8^Hunan Key Laboratory of Brain Homeostasis, Central South University, Changsha, China

**Keywords:** cervical radiculopathy, percutaneous laser discectomy, coblation, minimally invasive, clinical outcome, radiological

## Abstract

**Background:**

Minimally invasive techniques, such as percutaneous low-power laser discectomy (PLLD) and low-temperature plasma radiofrequency ablation (coblation) can be applied to treat degenerative cervical radiculopathy. However, less evidence supports the superiority of distinct minimally-invasive therapy. Our study aimed to evaluate the clinical and radiological characteristics of the PLLD and coblation for cervical radiculopathy.

**Methods:**

This was a prospective, multicenter, cohort study (ChiCTR-ONC-17010356). The modified Macnab criteria was performed to assess the clinical improvement pre- and post-surgery. To evaluate the radiological effect, the Pfirrmann grading system and disk herniation index were applied with MRI.

**Results:**

In this study, 28 patients were enrolled in the coblation group and 30 patients in the PLLD group. The mean good-excellent rate at 3-month follow-up was 82.1% for PLLD group, and 66.7% for coblation group, respectively (*p* = 0.179). The PLLD group achieved higher good-excellent rate 6 and 12 months after discharge (92.9 vs. 70.0%, *p* = 0.026). Radiological data revealed that PLLD but not coblation treatment achieved significant reduction of disk herniation index (*p* < 0.0001). Coblation treatment did not change the Pfirrmann grades of cervical radiculopathy patients (*n* = 18), and 7 out of 17 (41.2%) patients achieved improvement after PLLD therapy. None obvious adverse event was observed in this study.

**Conclusion:**

Both PLLD and coblation are effective and safe option for patients with cervical radiculopathy. Better long-term clinical outcomes may be potentially associated with the improvement of disk degeneration after PLLD treatment.

## Introduction

Cervical radiculopathy is one common cause of chronic pain, resulting from the compression of cervical nerve root. It is estimated that the annual incidence of cervical radiculopathy is 107.3 per 100,000 for male, and 63.5 per 100,000 for female, respectively (1, 2). The characteristic syndrome of cervical radiculopathy typically includes upper extremity pain and, occasionally, sensorimotor deficits in the distribution of the affected nerves (3). In most cases, non-operative intervention is effective to provide relief from acute pain, such as immobilization, traction, medication therapy, physical therapy, and cervical steroid injection (4, 5). However, about one-third of patients with cervical radiculopathy are unresponsive to the conservative treatment. Surgical intervention may be considered after 6–8 weeks of conservative care.

Conventional open procedures achieve good or excellent clinical outcome in carefully selected population who are insensitive to non-surgical management (6). In addition to the conventional open surgery, minimally invasive procedures for discectomy may reduce both pain and structural damage (7–9). Various minimally invasive procedures, such as percutaneous laser discectomy, low-temperature plasma radiofrequency ablation (coblation), and endoscopic discectomy are alternative options for patients who refuse to undergo open procedure (10–12). Several systematic reviews have demonstrated the effectiveness of these minimally invasive procedures. However, no evidence supports the superiority of each approach in the treatment of cervical radiculopathy.

Percutaneous laser discectomy can ablate and vaporize the nucleus pulposus with laser power. Compared with conventional microdiscectomy, similar clinical outcome was observed in the laser therapy for sciatica (13). Low to moderate power (1–5 W) continuous wave (CW) laser radiation can be delivered to herniated disk tissue at wavelength of 970 nm (14). Compared with the conventional laser device (power over 10 W), percutaneous low-power laser discectomy (PLLD) may be a safer option. To avoid potential damage of surrounding tissue, coblation nucleoplasty may be a safe approach for the relatively low central temperatures (40–70°C) during procedure (15). Additionally, heat radiation decreased from 40 to 20 to 0°C when the radius to the probe tip extended from 0 to 1 to 5 mm (16). Thus, the therapeutic and radiological effect may vary based on the strategy of minimally invasive procedure. In this study, we aimed to assess the clinical and radiological outcomes of patients with cervical radiculopathy, treated by PLLD or coblation.

## Methods

### Study Design and Participants

**Figure 1:**
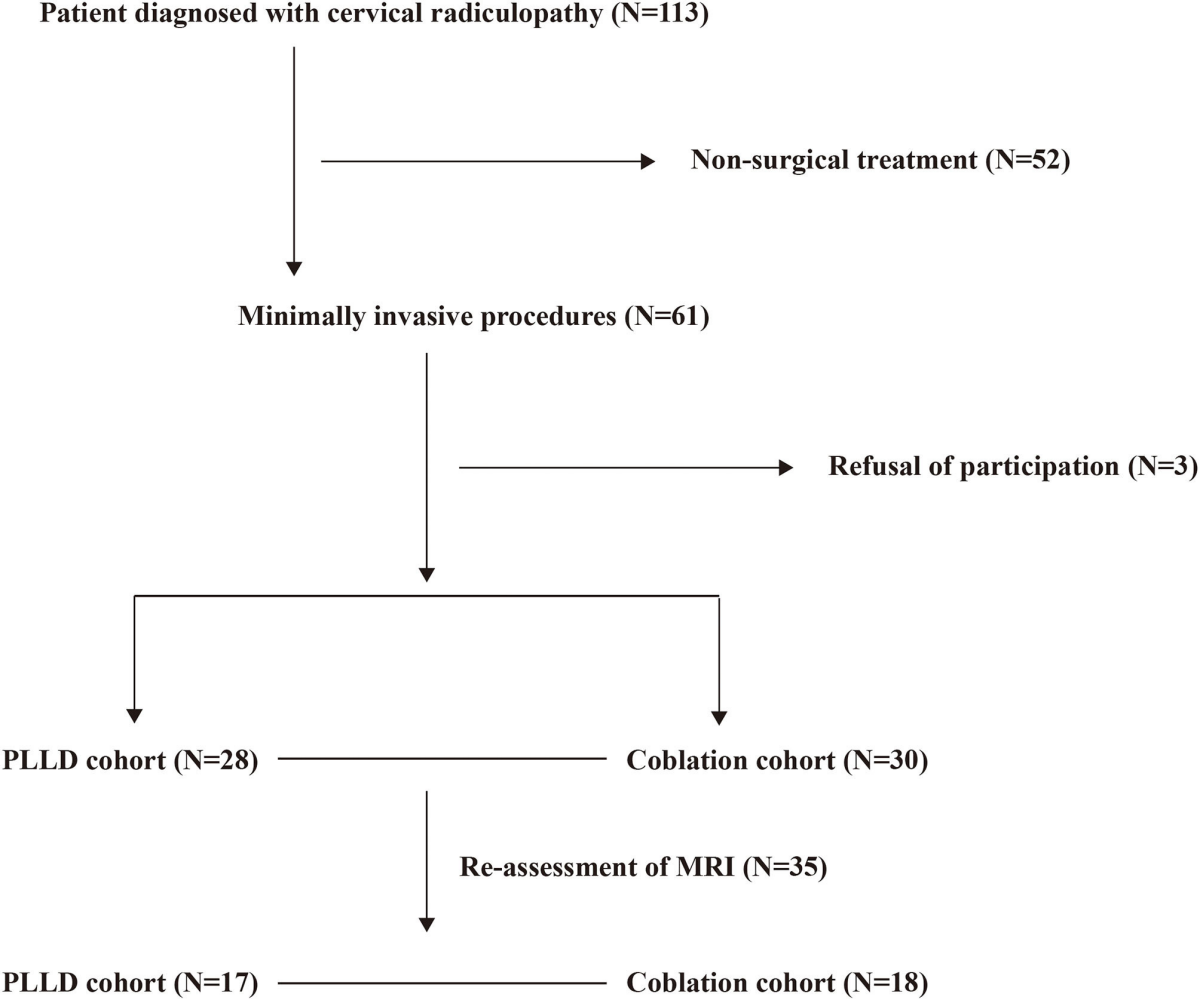
The flow chart of patient selection.

This was a prospective, multicenter, cohort study. Centers of pain medicine from four tertiary hospitals in Hunan Province, China, were invited to participate. All patients who met the enrollment criteria and were willing to take part in this study, were enrolled consecutively (from August 2015 to December 2017) to reduce the selection bias. All participates received routine care and therapy upon admission according to each hospital protocol to reflect the real practice environment. All patients were invited for the clinical follow-up at 3-, 6-, and 12-month after operation. The study was conducted in accordance with the Declaration of Helsinki and approved by the Ethics Committee of The Third Xiangya Hospital, Central South University (2016-S240). Written consent was acquired from all participates in this study. The study was registered at http://www.chictr.org.cn (ChiCTR-ONC-17010356). The selection of participants is given in the (Figure 1).

### Eligibility Criteria

Patients were enrolled if they fulfill the criteria as follow: (1) aged between 20 and 80 years; (2) diagnosed with cervical radiculopathy according to history, physical examination, imaging, and additional tests (17); (3) presented with moderate to severe pain (Visual Analog Scale over 4 of 10) after conservative therapy; and (4) unwilling to undergo the conventional open surgery.

Patients were excluded from this study if imaging test indicated cervical spinal instability, severe cases of spinal stenosis, annulus fibrosus calcification, and ossification of the posterior longitudinal ligament. Patients with severe co-morbidity who may not undergo the surgery were excluded from this study.

### Surgical Techniques

**Figure 2:**
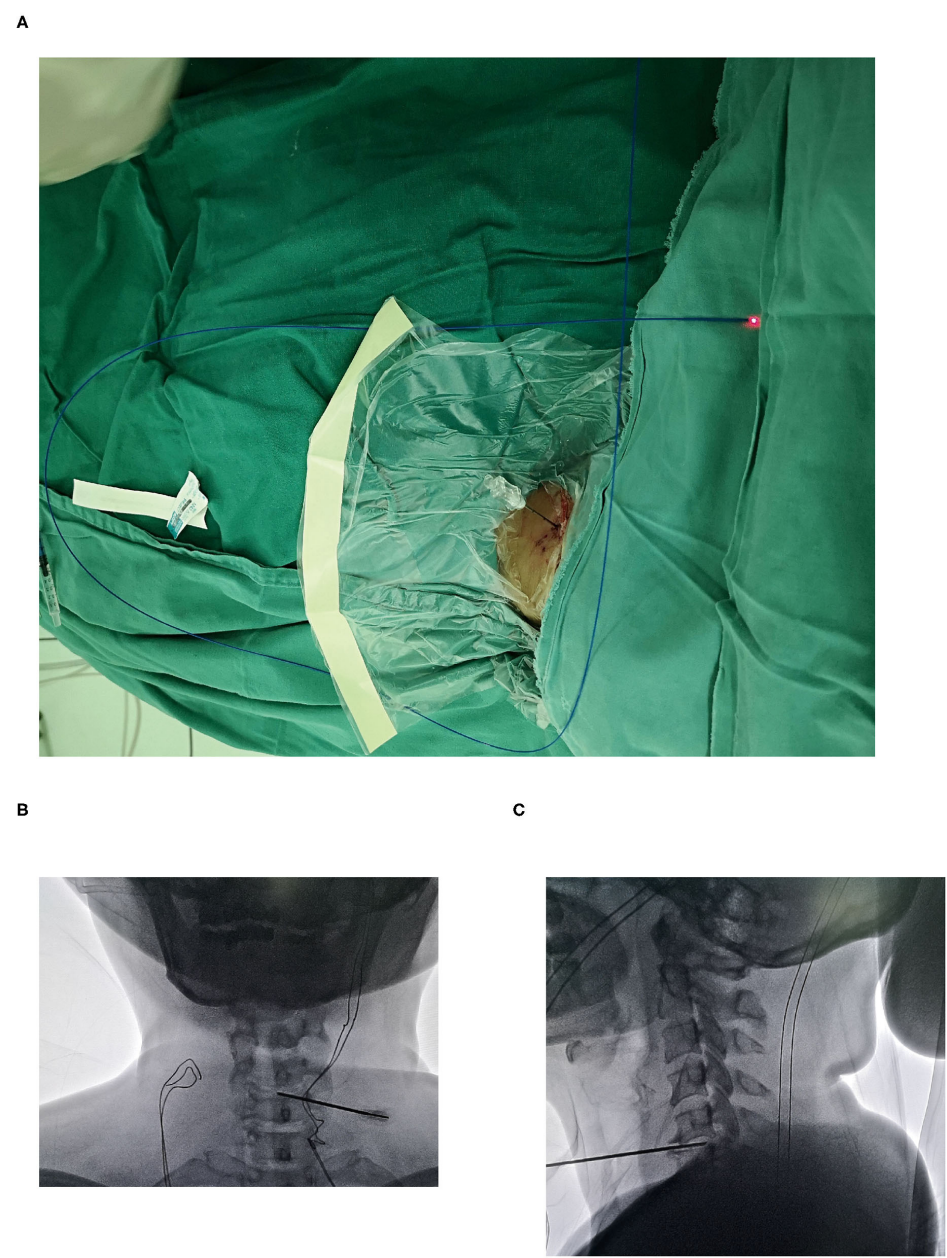
Placement of cannula during minimally invasive procedures. **(A)** Introducing cannula inserted in a 30 degrees angle medially with radiological guidance. Cannula position was confirmed by the **(B)** anterior-posterior and **(C)** lateral view.

The technique of minimally invasive procedure was left to the preference of the surgeon, either by PLLD or coblation. The procedure was performed under fluoroscopy by using a C-arm unit, with the patient placed in a supine position. Procedures of PLLD and coblation were conducted similarly in an anterior approach (11, 13). The shoulders of patient were stabilized to achieve better visualization of the lower cervical disk. To facilitate the access to the intervertebral disk space, head and neck were slightly hyperextended during surgery. Operation was performed under local anesthesia, with a solution of 1% lidocaine infiltration into the skin and subcutaneous tissue. Light sedation was administrated if necessary. The vital structures (trachea and carotid artery) were palpated away from the surgical access to avoid potential damage. One 19-G cannula with an internal mandrel was positioned against the anterior surface of the annulus fibrosus. The cannula was inserted under the guidance of lateral view of fluoroscopic imaging. The cannula tip was positioned at the distal third segment of disk as shown in (Figure 2). The parameter of surgical device is described as below.

For coblation, the mandrel was then replaced by the electrodes, connected to one bipolar radiofrequency-based system (SM-D380C; Gaotong, Xian, China). An electrical field was created to active the electrolytes and molecules to generate a field of plasma. The resultant plasma field dissolved a small amount of tissue within the targeted tissues at relatively low temperature (40–70°C). After 4–5 s of ablation, the electrode was repositioned to another part of nucleus according to the topography of the herniation. The duration of coblation at single cervical disk ranged between 5 and 10 min.

To perform laser discectomy, one optic fiber (diameter of 200-μm) was inserted through the cannula. The distal ending of fiber was plugged into one laser generator (Alaude-01; Keheng, Heilongjiang, China). The amplitude of laser was set at 2 W with 1 s width of pulse. The output of laser ranged 150–200 J for the first location. The cannula was pulled out slightly to the middle line of disk to apply another laser ablation. The total out of laser was no more than 350 J per disk. One key step of the procedure was to inject a small amount of saline (1–2 ml) into the disk through the cannula between the interval of pulse. The laser vaporized the nucleus pulposus with high temperature to achieve decompression of nerve root, and small air bubble can be observed when laser initially administrated. It took about 10 min to perform the laser discectomy for each cervical level.

### Postoperative Care

One prophylactic treatment of Cefuroxime was applied intravenously 30 min prior to surgery. The analgesic non-steroidal anti-inflammatory drug was administrated routinely in next 2 days (intravenous Flurbiprofen 100 mg per day). The patients were told to stay in bed for at least 2 days. To avoid the potential damage due to disability of cervical spine, a rigid neck collar must be wearing in the following month after discharge.

### Clinical Evaluation

The clinical outcome was defined as excellent, good, fair, no improvement, or worse, based on the modified Macnab criteria (Table 1) (18). The visual analog scale (VAS) ranging from 0 (pain free) to 10 (worst pain imaginable) was used to evaluate the overall severity of pain when admission.

**Table 1:** Clinical outcome assessed by the modified Macnab Criteria.

**Classification**	**Criteria**
Excellent	Free of pain; no restriction of mobility, normal work, and activities.
Good	Occasionally non-radicular pain. Relief of presenting symptoms. Able to return to modified work.
Fair	Partial improvement of functional capacity, still handicapped or unemployed.
Poor	None or insufficient improvement of objective symptoms with root involvement, further operative invention needed.

### Radiological Measurement

**Figure 3:**
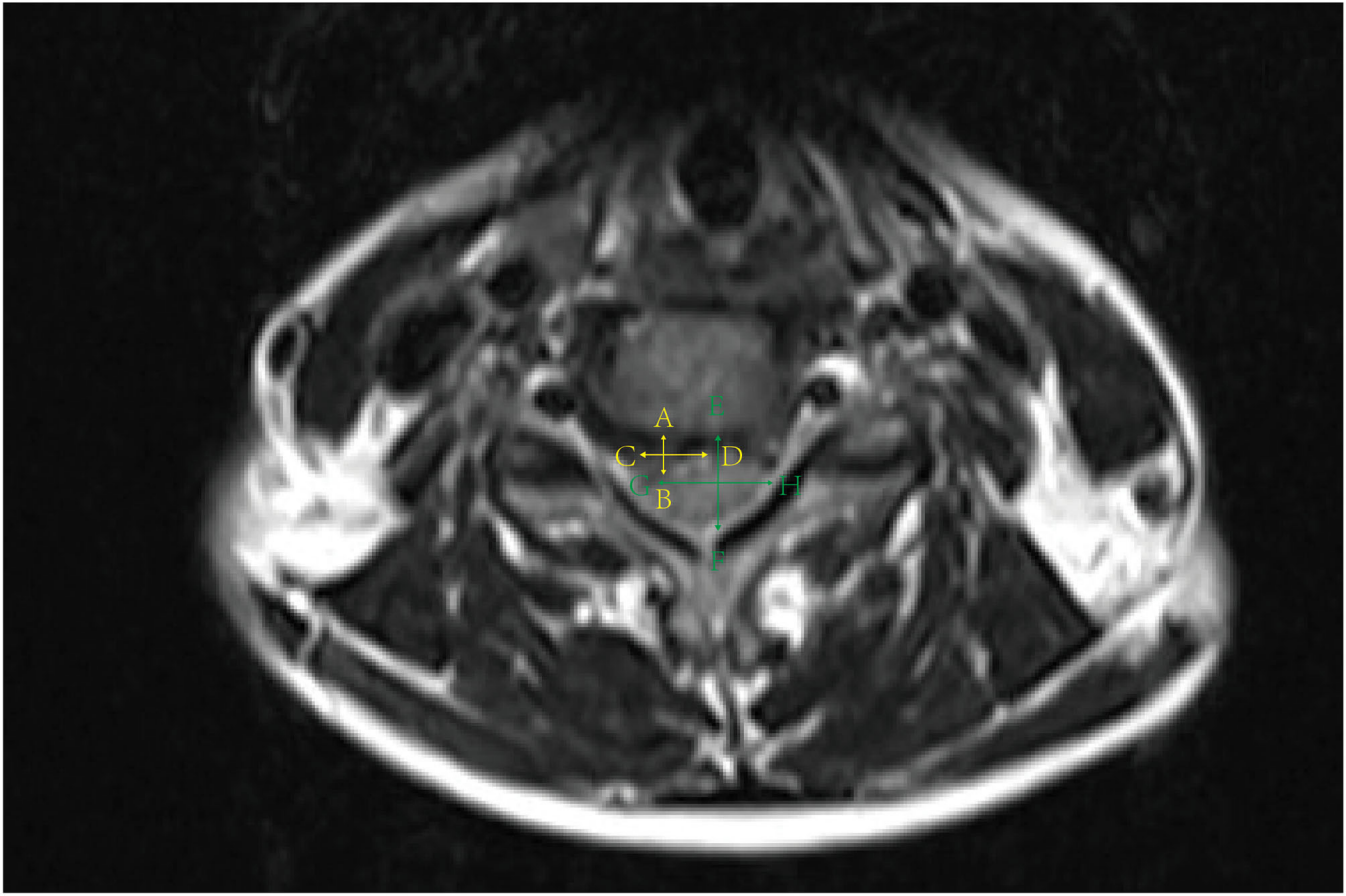
Measurement of disk herniation index.

The disk herniation index was determined by using MRI (Ingenia 3.0T, Philips Healthcare, The Netherlands). Detail of disk herniation index measurement has been described by Kang et al. (19). Briefly, we measured the disk and intervertebral foramen at intercept of the axial direction. The maximum anteroposterior herniated disk length was recorded as (AB), and that of canal was marked as vertebral foramen length (EF). The width of herniated issue (CD) was recorded as the distance at level of middle line of herniated disk length (AB). The width of the spinal canal was calculated at the same level (GH). The calculation of disk herniation index was ([AB × CD]/ [EF × GH]) ×1,000, as shown in (Figure 3).

To evaluate the changes on disk degeneration, Pfirrmann grading system was applied on routine T2-weighted MRI (20). Lower grades were associated with improved disk degeneration as shown in (Table 2).

**Table 2:** Summary of the Pfirrmann grading system for evaluation of disk degeneration.

**Grades**	**Structure**	**Distinction of nucleus and annulus**	**Signal intensity**	**Height of intervertebral disc**
I	Homogenous, bright white	Clear	Hyperintense, isointense to cerebrospinal fluid	Normal
II	Inhomogeneous with or without horizontal bands	Clear	Hyperintense, isointense to cerebrospinal fluid	Normal
III	Inhomogeneous, gray	Unclear	Intermediate	Normal to slightly decreased
IV	Inhomogeneous, gray to black	Lost	Intermediate to hypointense	Normal to moderately decreased
V	Inhomogeneous, black	Lost	Hypointense	Collapsed disc space

### Follow-Up

To assess the long-term therapeutic effect, patients were asked to visit the pain clinic 3, 6, and 12 months after surgery. Follow-up MRI imaging was performed at 6-month follow, and the imaging data were not obtained for all patients. Consequently, MRI was collected from 53% patients (31 out of 58).

### Statistical Analyses

Continuous variables are presented as the mean ± SD or median with 25–75th quartile range, and proportions for categorical variables. Independent samples *t*-test, Mann–Whitney *U*-test, Wilcoxon's test, or chi-square test of Fisher's was applied when appropriate. The value of *p* < 0.05 was considered significant. Data were analyzed with Prism version 8.0 (GraphPad, San Diego, CA, United States).

## Results

### General Demographics

Fifty-eight patients diagnosed with cervical radiculopathy were enrolled from four tertiary hospitals. About 52% (*n* = 30/58) patients undertook the coblation therapy, and 28 subjects for PLLD treatment, respectively. The average age was 56 years and the most common affected levels were C5/6 and C6/7 (52 out of 65 disks). All patients presented with moderated to severe pain, with mean VAS scores of 6.8 ± 1.0 before surgery. The pain severity decreased to 0.2 ± 0.5 after PLLD subgroup and 1.9 ± 1.8 in the coblation cohort, respectively. The demographic data of enrolled patients are given in the Table 3.

**Table 3:** Clinical and demographic data of patients with cervical radicular pain.

	**PLLD group**	**Coblation group**	***P*-value**
Number	28	30	
Ages (years)	56.0 ± 8.6	56.5 ± 11.3	0.87
Female gender (*n*, %)	13 (46.4)	13 (43.3)	>0.99
Duration (months)	5 (1–33)	2 (1–24)	0.32
**VAS**			
Baseline	6.7 ± 1.2	6.9 ± 0.7	0.38
Last Follow-up	0.2 ± 0.6	1.9 ± 1.8	<0.01
**Affected level (** ***n*** **, %)**			
C3/4	3 (10.0)	2 (5.7)	0.66
C4/5	3 (10.0)	5 (14.3)	0.72
C5/6	14 (46.7)	18 (51.4)	0.81
C6/7	10 (33.3)	10 (28.6)	0.79
Follow-up MRI	17 (60.7)	14 (46.7)	0.31

### Clinical Outcomes

**Figure 4:**
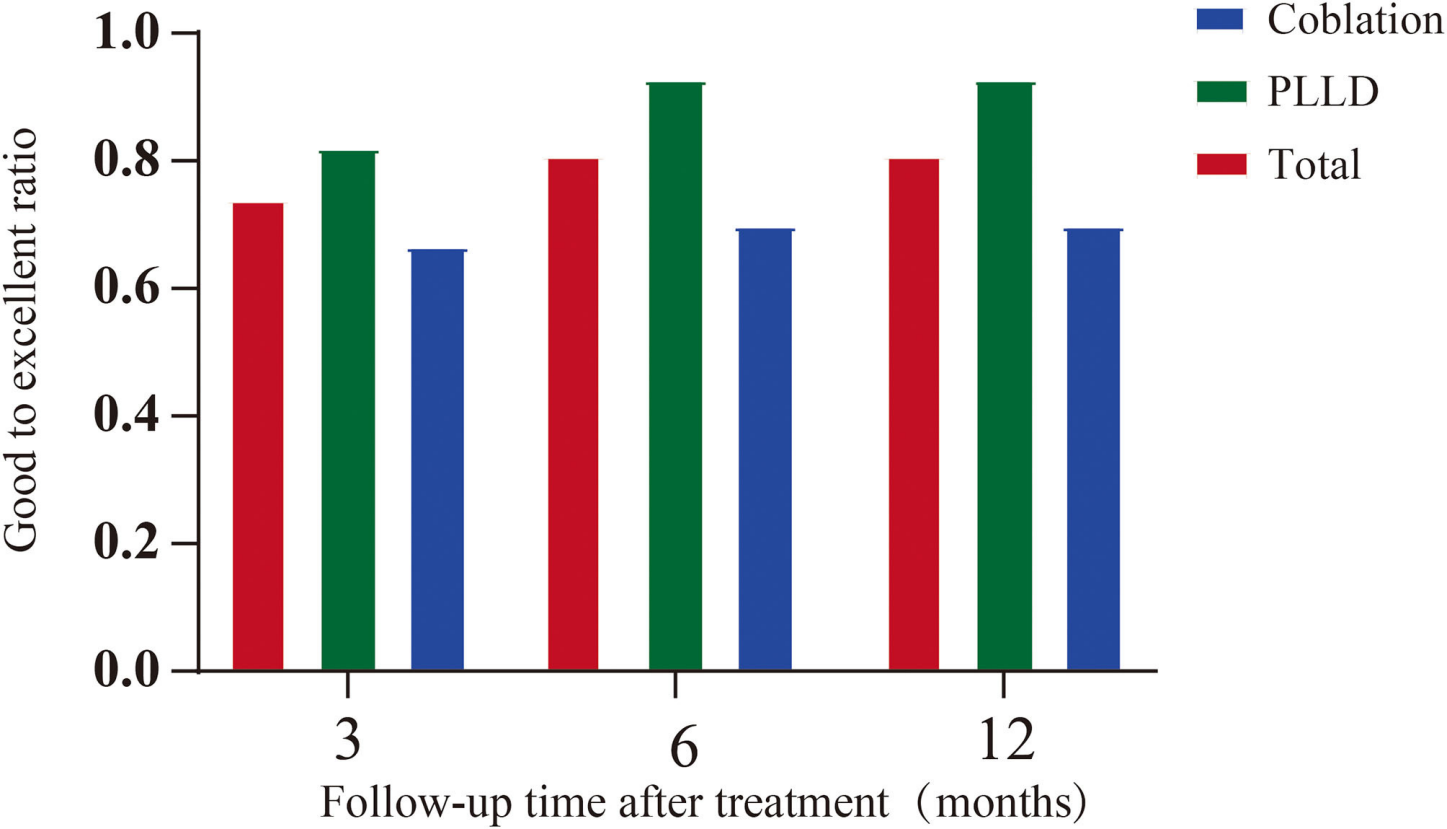
Clinical outcome after minimally invasive surgery assessed by the modified Macnab Criteria.

All 58 patients accomplished the 3-, 6-, and 12-month follow-up. According to the modified Macnab Criteria, clinical outcome could be judged as excellent or good in 82.1% patients (*n* = 23/28) with PLLD treatment, and 20 out of 30 (66.7%) with coblation at 3-month follow-up, respectively. The ratio of excellent or good outcome became significant between groups at 6- and 12-month visit (*p* = 0.026), as shown in (Figure 4). Only one patient (1.7%) who underwent coblation felt poor outcome of surgery after 6 months.

### Radiological Effects

**Figure 5:**
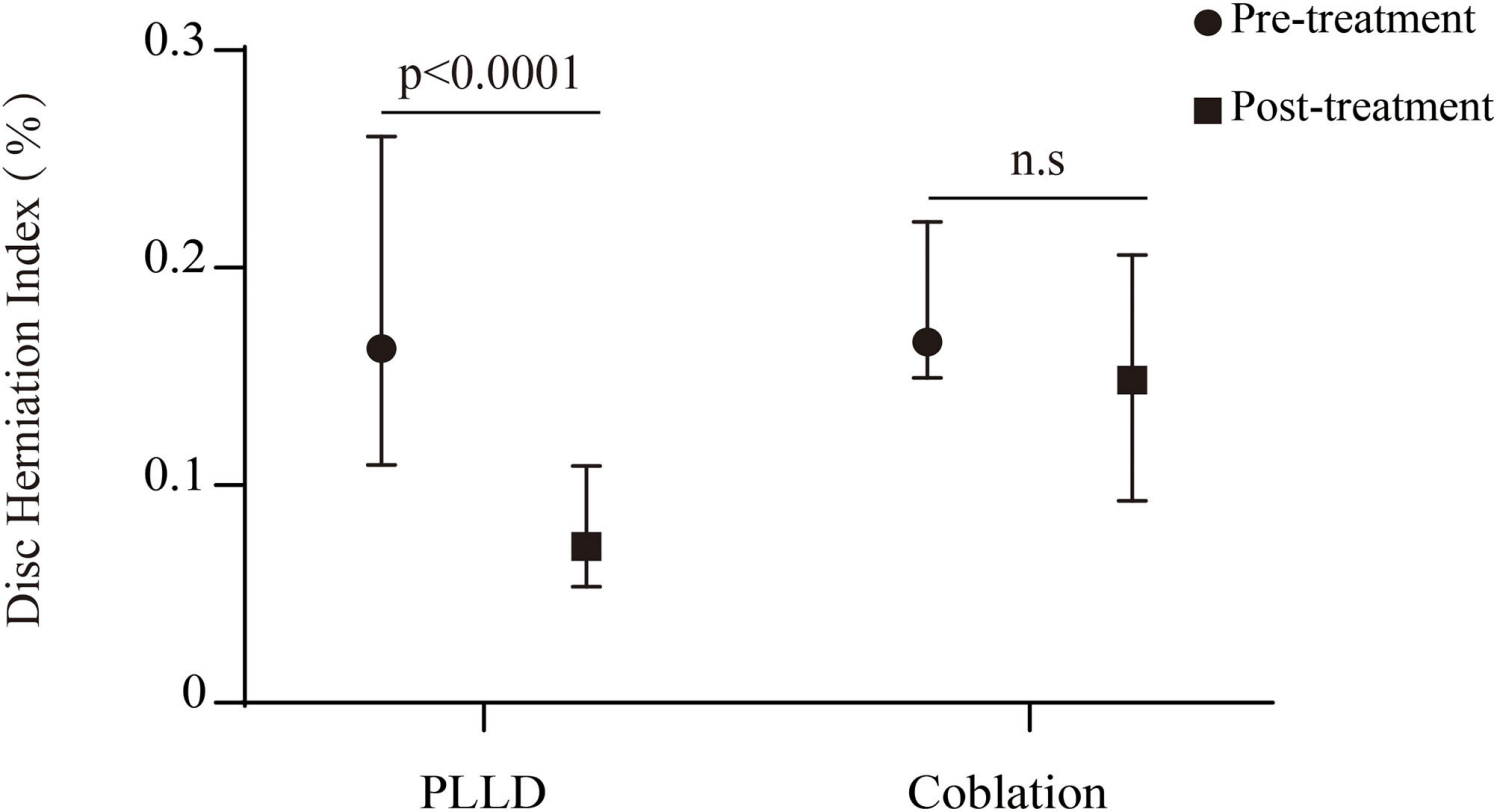
Radiological changes of disc herniation 6 months after therapy. n.s = the exact value is 0.1510.

A total of 31 patients took MRI follow-up at 6-month post procedure, with seventeen in the PLLD group. The disk herniation index in the PLLD group decreased significantly after surgery (*p* < 0.0001). However, we did not observe significant reduction of disk herniation within coblation cohort (Figure 5). Seven of 17 (41.2%) patients who underwent PLLD surgery presented improvement of disk degeneration according to the Pfirrmann grades tool as shown in (Table 4). The was no improvement in the Pfirrmann scores with coblation therapy.

**Table 4:** Comparison between pre- and postoperative disc degeneration.

	**Treatment (*n*, %)**	***P*-value**	
Improvement of Pfirrmann grades	PLLD	Coblation	
V → IV	2 (11.8)	0	
IV → III	4 (23.5)	0	
III → II	1 (5.9)	0	
Total number	7 (41.2)	0	<0.01

### Complications

No serious adverse events (e.g., headache, dysphagia, hemorrhage, infection, or cerebrospinal fluid leak) were observed in this study. Only one patient who took PLLD therapy presented with temporary trachyphonia after surgery. The symptom was relieved without any special intervention 2 days after surgery.

## Discussion

This prospective, multicenter, cohort study compared two minimally invasive procedures for the treatment of cervical radiculopathy. Previous publication reported that about 80–90% relief can be achieved *via* anterior cervical disk operations, such as percutaneous laser discectomy and low-temperature plasma radiofrequency ablation (21, 22). However, no evidence has supported the superiority for either approach. The clinical outcome is closely associated with the cervical disk degeneration, which is routinely assessed by the MRI (23, 24). In this study, we examined the relationship of clinical outcome with radiological changes by different minimally invasive procedures.

Despite the non-randomized design, our data demonstrated similar success rate with previous studies (25, 26). The overall good to excellent rate was 92.9% in PLLD group at 12-month follow, and 70% for coblation, respectively. Although it remains controversial whether minimally invasive procedure is superior to non-surgical treatment (27). In some cases, sufficient relief may not be achieved with conservative therapy (28). Similarly, the candidates of this study still presented with moderate to severe pain after at least 4-week conservative treatment.

To date, few studies compared the effectiveness of different minimally invasive procedures in the management of cervical radiculopathy. In this study, we found that the long-term therapeutic effect was inconsistent between different approaches. The short-term clinical outcome was not significant, and higher good to excellent rate was reported in the PLLD group 6 months after surgery. However, previous data demonstrated that better clinical outcome was achieved by coblation compared with radiofrequency thermocoagulation, possibly due to different population affected by lumbar degenerative pain (15). Symptomatic cervical disk herniation is associated with degenerative changes at adjacent and non-adjacent levels (29). Comparisons of disk degenerative status after laser or coblation intervention, however, must be taken into consideration for long-term clinical outcome.

**Figure 6:**
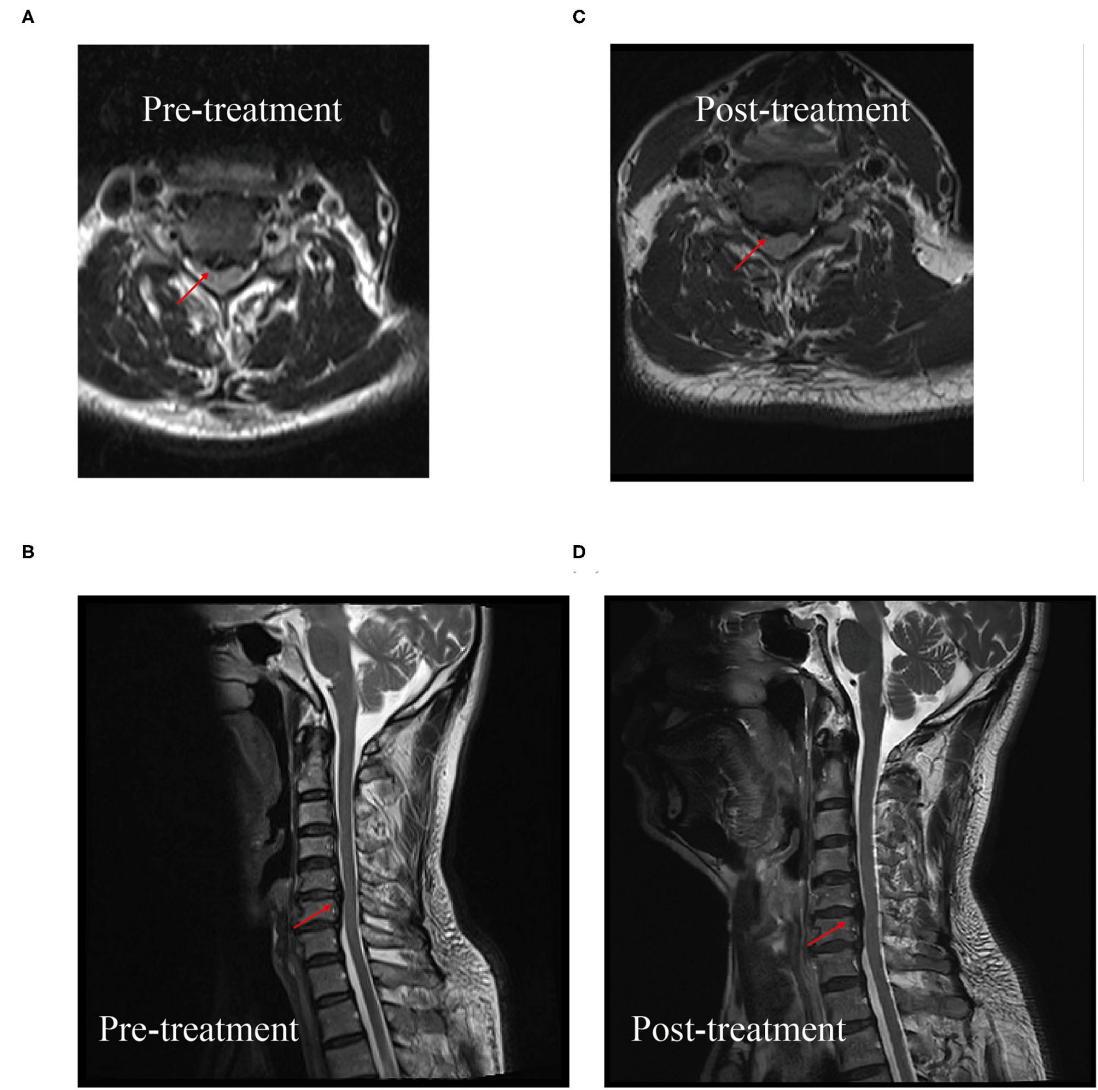
Comparison of MRI between pre- **(A,B)** and post-treatment **(C,D)** in a male patient who underwent coblation surgery. The red arrow indicates the cervical disc herniation in sagittal plane.

MRI has been most widely used to assess the spine degeneration for its high sensitivity to detect the water content of disk. Decompression of nerve root is the key for minimally invasive procedure, which can be measured as disk herniation index (19). The long-term relief from pain was associated with significant reduction of herniated tissues in PLLD group. The total laser energy was no more than 350 J/disk, much lower than the conventional laser output which may cause potential injury to the surrounding tissues (30, 31). Consequently, only one patient (3.6%) in PLDD cohort presented with temporary trachyphonia after surgery and recovered without any special intervention. The disk herniation index was not decreased significantly after plasma coblation therapy. The relatively low temperature during surgery may reduce the disk pressure without obvious change of herniation size. Based on T2-weighted imaging, a Pfirrmann grading tool was a reliable indicator of cervical disk degeneration (32, 33). In this study, we found that the PLLD treatment achieved significantly morphological improvement at 6-month follow-up. However, no improvement was observed after plasma radiofrequency therapy (Figure 6). This finding was consistent with previous quantitative research, about 17.6% of cases demonstrated progressive degeneration with coblation treatment (34).

The main limitation of this study is the nature of observational non-randomized design. Thus, we may not avoid the selection bias due to the distinct procedure approach, indication, co-morbidity, and general demographics of participants. Consequently, we may not exclude their impact on the clinical or radiological outcome. We think it is necessary to perform randomized, controlled clinical trials with larger sample size to further confirm the superiority of each method, and its effect on the degenerative development of cervical spine. Besides, a quantitative imaging study may be needed to further examine the degenerative changes after the minimally invasive procedures.

## Conclusion

Taken together, the present study demonstrated that both the PLLD and coblation are effective and safe minimally approaches for patients with cervical radiculopathy. Superior long-term clinical outcome of PLLD therapy may be potentially associated with the improvement of cervical degeneration.

## Data Availability Statement

The original contributions presented in the study are included in the article/supplementary material, further inquiries can be directed to the corresponding authors.

## Ethics Statement

The study was conducted in accordance with the Declaration of Helsinki and approved by the Ethics Committee of The Third Xiangya Hospital, Central South University (2016-S240). Written consent was acquired from all participates in this study. The study was registered at chictr.org.cn (ChiCTR-ONC-17010356).

## Author Contributions

DH, QL, and HZ designed this study. YHe, XW, CW, RHa, GG, RHu, XZ, JZ, and DH performed the surgeries. XL, ZW, and YN conducted the follow-ups. XL, ZW, YHu, ZL, and HZ analyzed the data. HZ wrote the manuscript. All authors contributed to the article and approved the submitted version.

## Conflict of Interest

The authors declare that the research was conducted in the absence of any commercial or financial relationships that could be construed as a potential conflict of interest.

## Publisher's Note

All claims expressed in this article are solely those of the authors and do not necessarily represent those of their affiliated organizations, or those of the publisher, the editors and the reviewers. Any product that may be evaluated in this article, or claim that may be made by its manufacturer, is not guaranteed or endorsed by the publisher.
